# Transcriptional network control of normal and leukaemic haematopoiesis

**DOI:** 10.1016/j.yexcr.2014.06.021

**Published:** 2014-12-10

**Authors:** Jonathan I. Sive, Berthold Göttgens

**Affiliations:** Department of Haematology, Cambridge Institute for Medical Research and Wellcome Trust and MRC Cambridge Stem Cell Institute, University of Cambridge, Cambridge, UK

**Keywords:** Haematopoiesis, Transcriptional regulation, Transcription factor

## Abstract

Transcription factors (TFs) play a key role in determining the gene expression profiles of stem/progenitor cells, and defining their potential to differentiate into mature cell lineages. TF interactions within gene-regulatory networks are vital to these processes, and dysregulation of these networks by TF overexpression, deletion or abnormal gene fusions have been shown to cause malignancy. While investigation of these processes remains a challenge, advances in genome-wide technologies and growing interactions between laboratory and computational science are starting to produce increasingly accurate network models. The haematopoietic system provides an attractive experimental system to elucidate gene regulatory mechanisms, and allows experimental investigation of both normal and dysregulated networks. In this review we examine the principles of TF-controlled gene regulatory networks and the key experimental techniques used to investigate them. We look in detail at examples of how these approaches can be used to dissect out the regulatory mechanisms controlling normal haematopoiesis, as well as the dysregulated networks associated with haematological malignancies.

## Highlights

• Transcription factor networks play a key role in determining cell fate in the haematopoietic system.
• Dysregulation of transcription factor networks is associated with haematological cancers.
• Genomic technologies and interactions with computational science are transforming our ability to investigate these networks.
• This review highlights recent examples of the role of transcription factor networks in normal and malignant haematopoiesis.

## Roles of transcription factors in cell differentiation

The haematopoietic system has long been at the forefront of research into the processes of normal and abnormal cell differentiation, both in foetal development and in the ongoing production of mature cells in adult life [1], [2]. Analysis of surface expression markers and isolation of distinct populations by flow cytometry has enabled the precise immunophenotypic characterisation of both developmental and mature forms of blood cells. Despite ongoing refinement, the “haematopoietic tree” outlining the major haematopoietic developmental pathways is well-defined in general terms with at least 14 mature cell types present in the normal adult, and a significant progress has been made into understanding the processes regulating these progressions [2], [3].

The key role of transcription factors (TFs) in regulating haematopoietic cell fate has long been recognised. Many TFs were originally identified following the observation of novel transcripts resulting from chromosomal translocations in haematological malignancies, for example Runx1 in the Runx1(AML1)–ETO fusion protein of t(8;21) acute myeloid leukaemia (AML) [4], Scl(Tal1) in t(1;14) T-cell acute lymphoblastic leukaemia (T-ALL) [5] and Lmo2 in t(11;14) T-ALL [6]. More recently, cellular reprogramming across haematopoietic lineages using combinations of TFs [7] and induction of pluripotency [8] has reinforced the key roles TFs play in determining cell fate.

An early attempt to provide a conceptual framework for cell fate decisions was made by Waddington, who conceived of an irreversible process described in terms of balls rolling downhill through bifurcating valleys which represented distinct maturing lineages [9]. The ability to manipulate these processes, with cells able to move between lineages under appropriate stimulation [10], [11], has suggested that a more plastic model more accurately represents reality in vivo. Using concepts from the network theory, Waddington׳s valleys can be replaced by “attractor states” – relatively stable conditions representing the totality of expressed genes at a given point, through which cells transit during differentiation [12]. Control of gene expression occurs through the combination of TFs, epigenetic regulators, and the cis-regulatory elements within the genome with which they interact (promoters, enhancers etc.) [13]. The totality of these components may be considered a “gene regulatory network”, and the inputs and outputs modelled as network motifs. Alon has described these in detail, and shown that motifs such as coherent and incoherent feed forward loops have demonstrable biological counterparts [14]. An example from haematopoiesis is the positive feed-forward loop formed by the Scl complex and Myb controlling the gene expression programme of T-ALL cells, as described below [15].

Just as normal haematopoiesis is determined by the effects of gene regulatory networks on gene expression, disturbances of these networks by mutations, deletions or oncogenic fusions of TFs can lead to alternative attractor states, representing malignant transformation. In the review below, we discuss experimental and analytical techniques for investigating normal and dysregulated gene regulatory networks in haematopoietic tissue, and go on to describe examples of these from the recent literature.

## Key experimental techniques in establishing TF networks

**Fig. 1:**
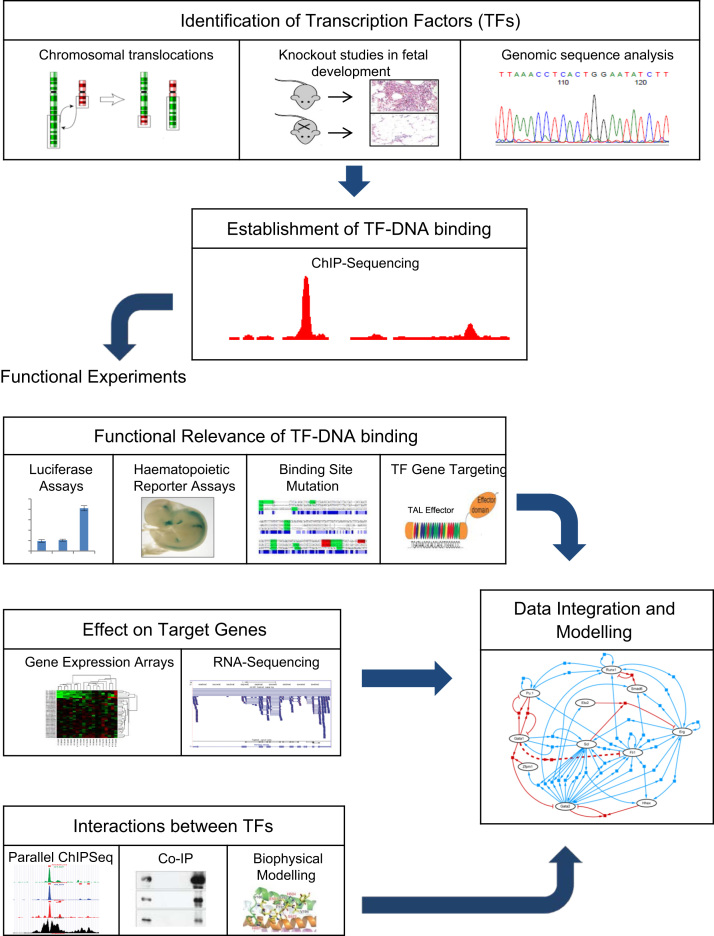
A schema for the investigation of transcriptional networks in the haematopoietic system.

Investigation into the roles of TFs in gene regulatory networks requires the identification of candidate TFs and their regulatory elements within a given system, and then confirmation of DNA binding and its effect on gene expression and cell phenotype. Interactions between multiple TFs may be suggested by the analysis of parallel DNA-binding experiments, and biophysical experiments can then identify individual components of TF-complexes and the interactions between them. Final integration of all the components and interactions increasingly requires bioinformatic analyses and computational network modelling; this can then be used iteratively to suggest new hypotheses for subsequent experimental validation. A schema illustrating this approach is shown in Fig. 1.

## Identification of transcription factors

As noted above, initial identification of many TFs resulted from observed gene-fusions produced from chromosomal translocations in haematological malignancies [4], [5], [6]. Knockout studies such as those on Scl in foetal development, can then determine the relevance of a given TF in haematopoiesis [16], [17].

Following the completion of the human genome project, it has been possible to identify putative TFs by sequence analysis for known DNA-binding motifs in coding regions, and subsequently assess the expression of these transcript sequences in different cell types. Dedicated online resources for the blood system, including gene expression compendia such as Gene Expression Commons [18], have further improved this process.

## Establishment of TF–DNA binding

A number of techniques may be used to establish the DNA-binding capability of a given TF, for example electrophoretic mobility shift assays (EMSA) [19], [20] and DNase footprinting assays [21]. The current “workhorse” of TF–DNA binding however is chromatin immunoprecipitation (ChIP), which produces a pool of DNA fragments enriched for the binding of the TF under investigation. Individual genomic regions can be interrogated using quantitative PCR (qPCR), but new sequencing technologies now allow global assessment of entire genomes using ChIP-Seq (ChIP followed by whole genome massively parallel DNA sequencing). Mapping of individual sequencing reads onto a reference genome allows an unbiased assessment of genome-wide binding events, with high resolution, low noise levels, and a high dynamic range. This technology represents a significant improvement over ChIP followed by array based hybridisation (ChIP-chip) [22], [23]. As with gene expression [18], [24] and epigenomic data [25], there are ongoing efforts to generate online resources that compile multiple ChIP-Seq studies generated by the haematopoiesis research community [26], [27].

Physical interactions between distal cis-acting regulatory elements and specific gene promoters can be mapped using Chromosome Conformation Capture (3C) methods and more recent variants such as 4C, 5C and Capture-C [28], [29], [30]. These techniques involve digestion and re-ligation of fixed chromatin followed by quantification of ligation junctions, which reflects the frequencies of interaction. Distal regulatory elements are commonly found many kilobases – and sometimes more than a megabase – away from the promoters they interact with, with one or more genes in-between the regulatory element and its actual target promoter. Chromosome Conformation Capture techniques have the potential therefore to address potential ambiguities in assigning distal elements to their cognate target promoters, and thus help refine our understanding of complex networks. Limitations persist however, in particular distinguishing genuine interactions from background noise, and ensuring that coverage is comprehensive enough to cover all relevant genomic interactions.

## Functional relevance of TF–DNA binding

Once binding sites have been identified, their functional relevance as cis-regulatory elements can be assessed by luciferase assays [31], [32], [33], [34], and their tissue-specific in vivo activity by reporter assays in transgenic mouse embryos [35], [36], [37]. Loss of function analysis through deletion of regulatory sequences in their endogenous genomic context, has been very cumbersome until recently. However, novel gene-targeting technologies such as TAL Effectors and CRISPR are now showing promise in assessing the effects of targeting specific regulatory elements in a more efficient manner [38].

TFs can either enhance or inhibit transcriptional activity, with many TFs able to act both as activators and repressors depending on the specific gene regulatory context (co-bound TFs, cell type, isoform, post-translational regulation etc.). The effect of a TF on a given gene regulatory sequence may be assessed by performing reporter assays, following the targeted mutation of specific binding sites [39], [40].

## Effect on target genes

Since the primary effect of TF activity is on gene expression, regulatory effects can also be evaluated on a genome-wide scale using expression arrays or RNA-Seq, following either overexpression or knock-down of a given TF. However, expression analyses cannot pinpoint the specific regulatory elements mediating transcriptional regulation, and indeed will also report expression changes due to indirect regulation. Recent advances now allow assessment of gene expression in individual cells, and these techniques have the potential to examine effects within small populations, such as those seen at the earliest stages of blood stem cell differentiation [41], [42]. Our group has used these techniques to confirm known interactions at the single cell level, and also identified new networks with previously unknown interactions, such as that between Gata2, Gfi1 and Gfi1b [43].

## Interactions between transcription factors

Interactions between different TFs may be suggested by genomic co-localisation on parallel ChIP-Seq experiments. Physical interactions can be established by co-immunoprecipitation (CoIP) assays, or if multiple factors are bound within a complex, more elaborate techniques such as high-stringency purifications [44], or SILAC [45], followed by mass-spectrometry and immunoblotting to establish the constituent factors. Yeast 2 hybrid assays have also been employed to confirm the functional relationships between TFs and cooperating proteins [46].

## Data integration and modelling

Translating experimental data into network models can be done using broadly “bottom-up” or “top-down” approaches. “Bottom-up” studies use small sets of TFs and regulatory elements to build up a detailed understanding of a given network. For example, the Scl–Gata2–Fli1 triad discussed below was initially investigated with detailed experiments, that were later complemented by computational modelling approaches [47], [48], [49], [50]. The increasing use of genome-wide datasets lends itself to “top-down” approaches, where advanced statistical analysis and computational modelling are used to infer networks, which can then be further investigated with specific, directed experimental work. Examples of this approach include the discovery of a “heptad” of seven co-binding TFs emerging from parallel ChIP-Seq experiments discussed below [37], and the central role played by c-Myc in B cells derived from comprehensive analysis of gene expression array and ChIP-chip data [51].

Integrated analysis using data generated by different research groups is also becoming increasingly feasible. The ENCODE project achieved this using a coordinated approach with pre-set experimental standards arranged between a number of groups [52]. More recently, post-hoc curation of existing datasets have yielded large tissue-specific collections [27], [53].

## Reconstruction of regulatory networks – normal haematopoiesis

Using the experimental and analytical techniques described above, several different gene regulatory networks have been delineated within the haematopoietic system. In addition to the examples discussed below, other groups have described networks in T-reg differentiation and function [54], B-cell lineage commitment [55], [56], and the Pu.1/Notch interaction determining T-cell or myeloid lineage fate [57].

## Pu.1/Gata1 antagonism

**Fig. 2:**
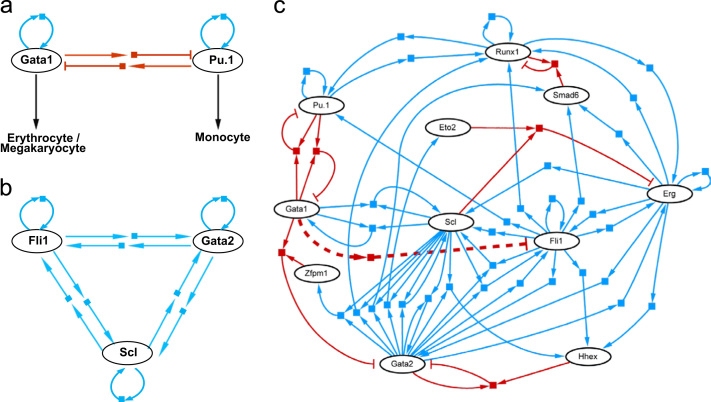
Modelling transcriptional regulatory networks in haematopoiesis. Activating interactions are shown as blue arrows, repressing interactions in red. (a) Cross-antagonism between Gata1 and Pu.1 determining erythroid–myeloid lineage fate. (b) The HSC triad composed of Fli1, Gata2, and Scl. Positive auto-regulatory activity between the three constituent factors maintains a bistable network. (c) Regulatory network based on ten TF ChIPSeq, illustrating interactions between constituent members of Scl/Lyl1/Lmo2/Gata2/Runx1/Erg/Fli1 heptad. The predicted–and subsequently validated–negative regulation of Fli1 by Gata1 is shown by a dashed line.

An example of mutual antagonism between TFs determining lineage commitment is the interaction between Pu.1 and Gata1, the study of which illustrates the beneficial interactions of both experimental and analytical work (Fig. 2a). Pu.1 expression in the erythroid–myeloid lineage causes monocytic differentiation, while Gata1 causes erythroid and megakaryocytic differentiation [58]. Both TFs positively auto-regulate their own expression [59], [60], and Gata1 has been shown to reduce Pu.1 expression by binding at its regulatory regions [61], as well as directly inhibiting Pu.1 protein activity at the Ets-domain [62]. Wontakal et al. used mathematical modelling to demonstrate that the combination of mutual inhibition, and repression of opposing downstream targets maximises the antagonistic interaction between the two TFs [63].

An alternative modelling approach to this system suggested the requirement for a previously unidentified third element to interact between Pu.1 and Gata1 to provide bistability, a desirable feature for robust lineage determination [64]. Subsequent discovery of the role of Tif1g in differentially controlling the levels of Gata1 and Pu.1 in HSCs, makes this is an attractive candidate for this role [65].

This model has recently been questioned by single cell expression data from our group, which showed that while the majority of progenitor cells expressed only either Pu.1 or Gata1 (consistent with mutual inhibition), a small number expressed both TFs with apparently positively correlated levels (not consistent with mutual inhibition). This illustrates the power of single cell analysis to shed new light on regulatory networks, something that is likely to become more prominent in the future [43].

## Scl/Gata2/Fli1 triad

An example of a triad of cross-regulatory TFs is provided by the interactions of Scl, Gata2 and Fli1 in foetal haematopoiesis (Fig. 2b). Initial studies on the Scl+19 enhancer region revealed binding by Fli1 and Gata2 via Ets and Gata motifs respectively [47]. Computational genome-wide searches subsequently revealed the same cluster of binding-motifs at the Fli1+12 [48] and Gata2–3 enhancer regions, with evidence of haematopoietic-localised activity demonstrated by transgenic mouse embryo assays in each case. Positive regulatory activity between each of the three TFs was shown by targeted mutation of DNA-binding sites followed by luciferase assays [49]. Subsequent modelling of this co-regulatory system revealed a network that is both bistable (either “all on” or “all off”), and characterised by low-pass filtering (i.e. relatively insensitive to low level perturbations from other factors such as Notch and Bmp4) [50].

## Scl/Lyl1/Lmo2/Gata2/Runx1/Erg/Fli1 heptad

The ability to integrate data from multiple TF ChIP-Seq experiments, has led to new insights into complementary binding patterns. An example is the ten TFs tested in a murine multipotent progenitor cell line, in which a statistically overrepresented binding pattern identified a “heptad” of combinatorial binding by seven TFs (Scl, Lyl1, Lmo2, Gata2, Runx1, Erg and Fli1). The absence of binding motifs in a significant proportion of TF-bound regions for some of the factors suggested protein–protein interactions, which were confirmed by CoIP experiments (e.g. Runx1 and Scl) [37]. Subsequent research has confirmed a similar binding pattern of the TF “heptad” in human primary CD34+ cells [66]. Boolean modelling of the original dataset predicted patterns of gene expression which matched those of known haematological cell states. Looking in detail at the network suggested a “missing link” in the interactions determining erythroid differentiation, which was hypothesised to be negative regulation of Fli1 by Gata1. Subsequent experiments validated the ability of Gata1 to negatively regulate expression of Fli1 by binding at its enhancer, confirming the predicted interaction and supporting the value of this type of network analysis (Fig. 2c) [67].

A further study used a gene expression signature based on the heptad binding pattern to stratify human AML expression datasets, where interestingly it was identified as an independent risk factor for poor prognosis [68]. This suggests that the net effect of the combinatorial binding of these seven TFs may be to maintain a stem cell “attractor state” in leukaemic cells, with a direct impact on clinical outcome.

## Dysregulated networks in leukaemia

Given the tightly controlled interactions between TFs in determining and maintaining normal haematopoiesis, it is unsurprising that disturbances to these systems can lead to dysregulation, and ultimately the abnormal proliferation of cells characterising haematological malignancies. Three well-described examples are detailed below, but a number of other studies demonstrating TF dysregulation in haematopoietic networks have been performed, for example TLX1 and TLX3 fusion oncoproteins down-regulating the Runx1 promoter in T-ALL [69], Pu.1 and Irf9 antagonism of miR-342 expression following ATRA exposure in APML cells [70], and CEBPβ binding to Irf4, Xbp1 and Blimp1 regulatory sites in myeloma cell lines [71].

## Scl in T-ALL

Sanda et al., [15] investigated the regulatory network of Scl (Tal1), a TF that is overexpressed in a significant proportion of T-ALL cases [72]. Scl ChIP-Seq in T-ALL cell lines and primagrafts showed consistent binding patterns with E-box, Gata, Runx and Ets motifs identified within 200 bp of the Scl binding, and frequent regions of overlap between Scl and the TFs E2A, Heb, Lmo1, Lmo2, Gata3 and Runx1. Interdependence between Scl, Runx1 and Gata3 was suggested by the presence of reciprocal binding at regulatory elements on loci by members of the complex in T-ALL cells, and reciprocal shRNA knockdown of each of the triad members caused down-regulation of the others, as well as inhibiting cell growth and promoting apoptosis.

Principal-component analysis of gene expression in 75 primary T-ALL and seven normal samples using the set of 238 genes bound by Scl and significantly downregulated after Scl knockdown, clearly distinguished the Scl overexpressing cases, confirming the pathological and clinical relevance of this network. Finally, the oncogene Myb – previously described to be overexpressed in T-ALL [73], [74] – was downregulated by knockdown of Scl, Runx1 and Gata3, and conversely knockdown of Myb led to downregulation of many Scl targets. This pattern of Myb regulation by Scl and concurrent regulation of Scl targets by Myb, suggests a role in reinforcing the regulatory circuit in a feed-forward loop, pathologically maintaining an aberrant attractor state in Scl-driven T-ALL. Other small subcircuits with possible relevance for T-ALL have been discovered by analysing transcriptional control mechanisms of Lmo2 [75], [76].

## Runx1–ETO in t(8;21) AML

The role of Runx1–ETO (commonly referred to as AML1–ETO) in t(8;21) AML is well recognised [77], where it is generally thought to act as transcriptional repressor through recruitment of NCOR and HDAC proteins [78], [79], [80] and reduction of histone acetylation [81]. Sun et al. used a variety of techniques to investigate the components of the Runx1–ETO containing TF complex (AETFC), and their roles in leukemogenesis [44]. AETFC was isolated using a high-specificity anti-ETO antibody followed by high-stringency buffer purification, and the components of the complex were then identified by a combination of SDS-PAGE, immunoblot and mass-spectroscopy. A number of TFs were identified within the complex, including CBFβ, Lyl1, Lmo2, Ldb1 and the E proteins HEB and E2A. The interactions between them were investigated by CoIP, and ChIP-Seq confirmed co-localisation at a number of binding sites. Knockdown of individual AETFC components (especially E2A and HEB) in Runx1–ETO9a-transformed splenic murine cells reduced the degree of leukemogenicity, suggesting a key role for the individual components of the complex.

Subsequent X-ray crystallographic analysis identified a novel interaction at the NHR2 domain of Runx1–ETO and a binding site (N2B) on the E protein HEB. Disruption of this interaction reduced cell-initiating potential in in vitro models, as well as reducing leukemogenicity and improving survival in a mouse model. This study illustrates the power of combining genomic technologies, with biophysical and in vivo experiments to delineate the specific physical structure underlying a TF interaction, and assessing the effects of its manipulation on disease outcomes in animal models.

## CM in AML inv16

Another well-characterised fusion oncogene is CBFβ-MYH11 (CM) formed by the chromosomal translocation inv(16), which is clinically characterised by the AML subtype M4Eo (eosinophilic). CM combines the DNA-binding stabilisation CBFβ subunit of the core binding factor (CBF), with the smooth muscle myosin heavy chain MYH11. The oncogenic fusion protein has been hypothesised to act as a dominant negative mutation affecting normal Runx1 transcriptional function [82], [83]. Mandoli et al., [45] performed ChIP-Seq in the inv16 cell line ME1, using antibodies against each of the two CM fusion partners as well as for Runx1 and Scl. CM binding sites were all co-localised with Runx1 reinforcing the suggestion that the CM-binding is Runx1-dependent. However in contrast to the overall Runx-binding, the majority of CM binding events were at promoter sites, indicating that additional factors contribute in guiding CM to promoter regions.

Using a proteomic approach, the authors were able to identify co-binding proteins, including various TBP-associated factors (TAFs) of which TAF7 was confirmed to interact with CM by CoIP. ChIP for TBP and RNAPII (proteins that from a stable complex with TAFs in a pre-initiation complex) showed co-localisation, and reChIP experiments confirmed CM interaction with these basal transcriptional activators. Further TF ChIP-Seq showed other TFs (e.g. Elf1, Fli1, Pu.1, Erg) co-localised with CM. CM binding was associated with increased acetylation and altered gene expression, with both increases and decreases in transcription seen at different loci, in contrast to the purely repressive function of CM previously reported [83], [84]. Potentially relevant to the leukaemogenic function of CM was the observation that a proportion of those aberrantly regulated genes are involved in self-renewal pathways. These experiments therefore point to the CM oncoprotein subverting the normal binding activity of Runx1 and other cooperating TFs, to alter the overall transcriptional status of the cell moving it into a leukaemic phenotype.

## Conclusions

The role of TFs in controlling haematopoiesis has long been recognised, but we are now increasingly able to understand how interactions between TFs are crucial in regulating normal cell fate, and conversely how specific network perturbations can lead to malignant phenotypes. Two factors in particular have led to this greater understanding: innovation in genomic technologies, and increased interactions between laboratory and computational science.

ChIP-Seq has been key in allowing the interrogation of TF-binding events on a genome-wide basis at an increasingly affordable price, and the effects on gene expression changes can be assessed by expression arrays or RNA-Seq. Cell number remains a limiting factor for ChIP however, with 5000–100,000 cells the lowest numbers reported for a TF-ChIP, even with additional amplification steps used prior to sequencing [85], [86]. This is in contrast to advances in transcriptomic technology, where changes in gene expression can now be analysed at a single cell level, providing major insights in heterogeneous and rare cell populations [41], [87].

The volume of data generated by these new types of experiments requires analysis beyond the remit of most laboratory-based scientists, and has led to increasing interactions with computational scientists for analysis and network modelling. These models can be used to direct laboratory experiments, which in turn can refine the models in a “virtuous circle” of hypothesis generation, experimental testing and data analysis.

As demonstrated in the examples above, we believe that this approach is ideally suited to the investigation of complex biological systems such as the gene regulatory networks that determine cell fate. The next few years should see further advances in our understanding of the control of cell differentiation both in haematopoiesis and other fields, and provide further insights into the mechanisms of oncogenesis.
